# Proteomic Analysis of Mitochondrial-Associated ER Membranes (MAM) during RNA Virus Infection Reveals Dynamic Changes in Protein and Organelle Trafficking

**DOI:** 10.1371/journal.pone.0117963

**Published:** 2015-03-03

**Authors:** Stacy M. Horner, Courtney Wilkins, Samantha Badil, Jason Iskarpatyoti, Michael Gale

**Affiliations:** 1 Department of Immunology, University of Washington, Seattle, Washington, United States of America; 2 Department of Molecular Genetics and Microbiology, Duke University Medical Center, Durham, North Carolina, United States of America; 3 Department of Medicine, Duke University Medical Center, Durham, North Carolina, United States of America; SRI International, UNITED STATES

## Abstract

RIG-I pathway signaling of innate immunity against RNA virus infection is organized between the ER and mitochondria on a subdomain of the ER called the mitochondrial-associated ER membrane (MAM). The RIG-I adaptor protein MAVS transmits downstream signaling of antiviral immunity, with signaling complexes assembling on the MAM in association with mitochondria and peroxisomes. To identify components that regulate MAVS signalosome assembly on the MAM, we characterized the proteome of MAM, ER, and cytosol from cells infected with either chronic (hepatitis C) or acute (Sendai) RNA virus infections, as well as mock-infected cells. Comparative analysis of protein trafficking dynamics during both chronic and acute viral infection reveals differential protein profiles in the MAM during RIG-I pathway activation. We identified proteins and biochemical pathways recruited into and out of the MAM in both chronic and acute RNA viral infections, representing proteins that drive immunity and/or regulate viral replication. In addition, by using this comparative proteomics approach, we identified 3 new MAVS-interacting proteins, RAB1B, VTN, and LONP1, and defined LONP1 as a positive regulator of the RIG-I pathway. Our proteomic analysis also reveals a dynamic cross-talk between subcellular compartments during both acute and chronic RNA virus infection, and demonstrates the importance of the MAM as a central platform that coordinates innate immune signaling to initiate immunity against RNA virus infection.

## Introduction

Compartmentalization of biochemical processes and signaling responses is important for regulation of basic cellular functions. Communication between organelles, which occurs at physical contact sites between organelles, regulates many basic cellular functions. These sites of interaction between organelles are often dynamic in nature and have been implicated in human disease [1]. One of these contact sites between organelles is located at the interface between mitochondria and the ER and is called the mitochondrial-associated ER membrane (MAM). The MAM is a subdomain of the ER that physically contacts mitochondria and is a central organizer of several metabolic processes, including calcium and apoptotic signaling, synthesis of phospholipids, cholesterol trafficking, and inflammasome signaling, as well as regulating mitochondrial morphology [2–7]. Dysfunction of the MAM at the level of increased MAM function and ER-mitochondrial communication has been implicated in the pathogenesis of Alzheimer’s disease [8].

Recently, we have shown that the MAM also organizes antiviral signaling responses during the innate immune response to RNA virus infection [9]. RNA virus infection of mammalian cells drives an antiviral signaling response that results in the induction of the innate immune response, including production of type I interferon and expression of hundreds of interferon stimulated genes that regulate virus replication and spread. Antiviral innate immune signaling begins when pattern recognitions receptors sense virally-derived pathogen-associated molecular patterns (PAMPs) to activate this antiviral signaling cascade. The intracellular nucleic acids sensors that sense RNA virus-derived PAMPs include the cytoplasmic RIG-I-like receptor (RLR) proteins RIG-I and MDA5 (reviewed in [10–12]).

Following activation, RIG-I and MDA5 interact with their signaling co-factor MAVS, a transmembrane protein with a diverse localization profile, being localized to mitochondria and peroxisomes, as well as the to the MAM [9,13,14]. The reason for this diverse localization is not completely understood, but it has been proposed that MAVS supports different cellular signaling pathways from these organelles [9,13]. Our previous studies have shown that during the antiviral response RIG-I gets recruited to intracellular membranes, including the MAM [9,15]. Further, we have shown that MAVS interacts with both RIG-I and TRAF3, a downstream antiviral signaling protein, on the MAM [9]. In fact, the MAM serves as an organelle platform to link peroxisomes and mitochondria during the antiviral response[9]. Therefore, the MAM functions as a key signaling membrane microdomain during the antiviral response.

The importance of the MAM for organizing antiviral responses is highlighted by the fact that the hepatitis C virus (HCV) innate immune evasion program takes place on the MAM [9]. HCV is a positive sense single-stranded RNA virus, and worldwide approximately 130–170 million people are chronically infected with HCV [16]. While HCV RNA is sensed by RIG-I, the subsequent downstream signaling from RIG-I, including activation of the transcription factor IRF3, is blocked during HCV infection by the actions of the viral NS3/4A protease [17–23]. The HCV NS3/4A protease prevents this downstream signaling through cleavage of MAVS, which releases MAVS from intracellular membrane-association and prevents MAVS oligomerization [20,24–27]. Previously we found that HCV NS3/4A cleaves the MAM-associated MAVS, but not the mitochondrial-associated MAVS [9], suggesting that MAVS on the MAM is important for antiviral immunity to HCV [28]. Therefore, our previous studies implicate the MAM as a key mediator of the innate immune response to RNA viruses.

Protein trafficking into the MAM during RNA virus-mediated activation of innate immunity, either by viral proteins or by proteins involved in innate immune signaling, could result in changes in mitochondrial metabolism and apoptosis, as well as more generally regulating antiviral innate immune signaling. Here, we globally assess the dynamics of protein recruitment into the MAM during RNA virus infection by identifying and characterizing the proteome of the MAM, ER, and the cytoplasm from mock and RNA virus-infected cells. We found that proteins and cellular pathways are dynamically recruited to and from the MAM during RNA virus infection. Furthermore, we identified novel MAVS-interacting proteins that could be regulators of the innate immune response to RNA virus infection.

## Materials and Methods

### Cell culture and viruses

Huh7 and Huh7.5 [21], Huh7-HCV K2040 replicon cells that propagate culture-adapted variants of the Con1 HCV (genotype 1B) subgenomic replicon RNA [29], and the non-neoplastic hepatocyte PH5CH8 cells [30] were cultured according to standard techniques. Sendai virus strain Cantell was obtained from Charles River Laboratory.

### Plasmids and transfections

The pMyc-Mavs [20] plasmid has been described previously. Plasmids encoding human VTN (NM_000638.3), LONP1 (NM_004793.2), and RAB1B (NM_030981.1) were obtained (Origene). PCR was used to amplify each coding sequence following by subcloning the resulting product in frame into the *Not*I and *Pme*I sites of the pEF-Tak expression vector resulting in Flag-tagged constructs [31]. The sequences of oligonucleotides used are available upon request. All DNA sequences were verified by DNA sequencing. DNA transfections were done using FuGENE 6 (Roche). IFN-β promoter luciferase assays were conducted as described at 24h following mock or SenV infection [21].

### Subcellular fractionation

MAM, mitochondria, and microsomes were isolated from cells using Percoll gradient fractionation [32]. In brief, cells were harvested in ice cold PBS and lysed by Dounce homogenization in Sucrose Homogenization Medium (0.25 M sucrose, 10 mM HEPES, pH 7.4) with protease inhibitors. Differential centrifugation was used to isolate the post-nuclear supernatant from nuclei and cellular debris. The total microsomal fraction and crude mitochondrial fraction were isolated by centrifugation at 10,300 x *g*. Following this step, the microsomal fraction (which consists of vesicles from smooth and rough ER, as well as membranes from the Golgi apparatus and plasma membrane), was isolated following centrifugation at 100,000 x *g*. The resulting supernatant from this spin is the “cytosol” fraction, and it was concentrated by using Amicon Ultra 15ml filters. The crude mitochondrial fraction was purified through a self-generating Percoll gradient, and the collected mitochondria and MAM fractions were further purified by centrifugation at 6300 x *g*. The MAM was then collected following centrifugation at 100,000 x *g*. Fractions were resuspended in Mannitol Buffer B (0.225 M mannitol, 0.5 mM EGTA, 5mM HEPES, pH 7.4). Equivalent amounts of protein from each fraction were analyzed by SDS-PAGE and immunoblot. Protein concentrations were determined by BCA assay.

### Immunoblotting

Cells were lysed in a modified RIPA buffer (10mM Tris [pH 7.5], 150mM NaCl, 0.5% sodium deoxycholate, and 1% Triton X-100) supplemented with protease inhibitor cocktail (Sigma) and phosphatase inhibitor cocktail II (Calbiochem). Following harvest, protein was subjected to SDS-PAGE, transferred to nitrocellulose membranes in a 25 mM Tris-192 mM glycine-0.01% SDS buffer and blocked in 5% milk-phosphate-buffered saline with 0.1% Tween-20 (PBS-T) buffer. After washing, membranes were incubated with species-specific horseradish peroxidase-conjugated antibodies (Jackson ImmunoResearch), treated with ECL+ (GE Healthcare), and imaged on X-ray film.

### Immunoprecipitation

For immunoprecipitation, cells were lysed in IP buffer (10mM Tris [pH 7.5], 150mM NaCl, 5mM EDTA, 0.5% sodium deoxycholate, 0.1% SDS, and 1% NP40) supplemented with protease and phosphatase inhibitors. Protein was immunoprecipitated with anti-Myc antibody and captured on Protein G Dynabeads (Invitrogen). Bound protein complexes were washed in 1XPBS and then eluted using SDS sample buffer (0.125M Tris-HCl pH 6.8, 4% SDS, 20% Glycerol, 0.004% Bromophenol Blue, 10% β-mercaptoethanol).

### Antibodies

The following antibodies were used for immunoblot analysis: anti-Flag M2 (1:1000 dilution; Sigma), anti-Myc (1:2000 dilution; Abcam and Santa Cruz), anti-tubulin (1:5000 dilution; Sigma), anti-Calnexin (1:1000 dilution; Stressgen), anti-Cox-1 (1D6) (1:1000 dilution; Invitrogen), and anti-FACL4 (1:200 dilution; Abgent).

### Electron microscopy

Cells were fixed in 2.5% glutaraldehyde and 2% paraformaldehyde in 0.1M phosphate buffer. Postfixation was done in 1% osmium tetroxide plus 0.8% potassium ferricyanide in 100 mM sodium cacodylate, pH 7.2. Cells were prestained in 4% uranyl acetate, rinsed dehydrated, infiltrated in 1:1 acetone:Epon 812, then with 100% Epon 812 resin, and embedded in the resin. 60- to 80-nm thin sections were stained in lead citrate, rinsed, and poststained in uranyl acetate. Electron microscopy was performed on a JEOL 1200 EX equipped with a Sis Morada CCD camera.

### Protein Separation and LC-MS/MS

Protein separation and LC-MS/MS were performed by P. Gafken (FHCRC). Samples were prepared with PPS Silent Surfactant (Protein Discovery Inc) based on the manufacturer’s recommended protocol and digested with trypsin (Promega, sequencing grade TPCK treated). Trypsin digestion was carried out in 50 mM ammonium bicarbonate buffer (pH 7.8) overnight at 37°C at a trypsin to protein ratio 1:50. Peptides were analyzed on an LTQ-Orbitrap mass spectrometer (Thermo Scientific). The reverse phase column consisted of a trap column (100 μm x 1.5 cm) of Magic C18AQ resin in an IntegraFrit (New Objective) connected inline to an analytical column (75 μm x 27 cm) of Magic C18AQ resin in a PicoFrit. The LC-MS/MS acquisition consisted of a full mass spectrum followed by up to 5 data dependent MS/MS spectra of the 5 most abundant ions. The full mass spectrum scan range was 400–1800 m/z and the instrument resolution was set to 60K. The data dependent scans were collected using the following settings: Repeat Count = 1, Repeat Duration = 30, Exclusion List Size = 100; Exclusion Duration = 45; Exclusion mass width = 0.55 m/z low and 1.55 m/z high. Charge state screening was used allowing analysis of +2, +3, and +4 and higher charge states while rejecting analysis of +1 and unassigned charge states. Each sample was analyzed in three technical replicates of a total of 1–2 μg of protein. The LC-MS/MS data were searched against the human IPI database (version 3.70). The data processing and database search were performed in CPAS (https://www.labkey.org/wiki/home/Documentation/page.view?name=ms2) with X!Tandem search engine. Peptide Prophet [33] was used to evaluate peptide assignment at FDR<5% and Protein Prophet [34] was used to group peptides into proteins. Only peptides that passed the 5% FDR threshold for Peptide Prophet were used for the grouping. The full proteomics data set is available in S1 Table.

### Proteomics data analysis

Data processing and statistical analysis was performed with R/Bioconductor. Protein relative abundance was computed as the sum of spectral counts for all peptides mapped to that protein. Spectral counts were then normalized across samples by quantile normalization and assessed for differential expression per organelle using the *limma* package [35,36]. Proteins were identified as differentially expressed in each organelle given a Benjamini-Hochberg adjusted p-value of < 0.05 and a fold change of at least 2.

## Results

### Proteomic analysis of subcellular fractions during RNA virus infection

**Fig 1:**
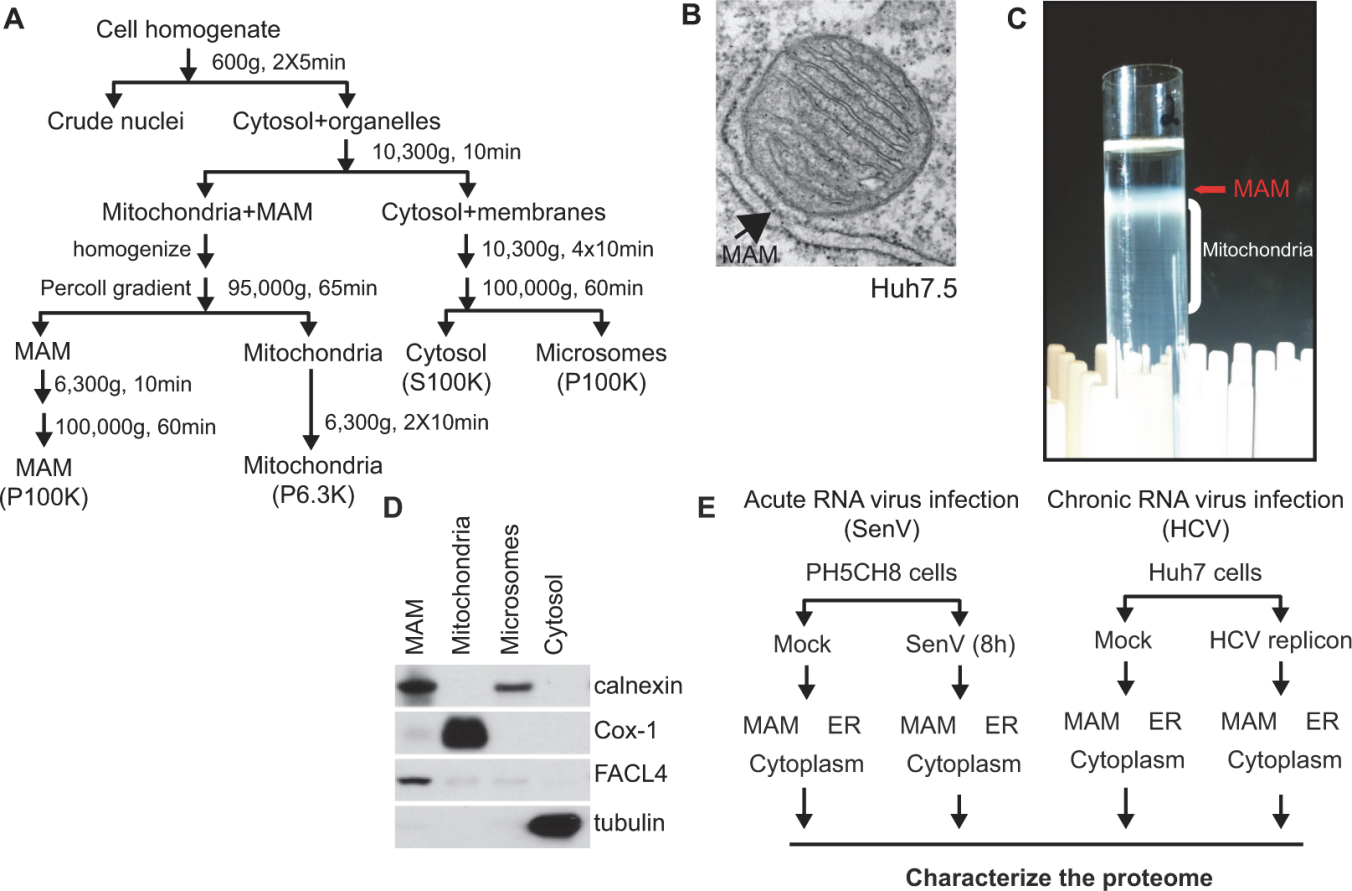
Experimental design for proteomic analysis of MAM fractions during RNA virus replication. (A) Percoll gradient biochemical fractionation scheme. (B) Electron micrograph of Huh7.5 cells. MAM is indicated by the arrow pointing to the membrane wrapping around a mitochondrion. (C) Percoll gradient illustrating a representative fractionation of PH5CH8 cells at the mitochondria/MAM isolation step. MAM and mitochondria were extracted from the gradient by a needle at the location indicated by the arrow and subjected to further purification. (D) Immunoblot analysis of biochemical fractions isolated from PH5CH8 cells. Fractionation markers: calnexin, ER; Cox-1, mitochondria; FACL4, MAM; tubulin, cytosol. (E) Experimental scheme for proteomic analysis.

Using an established Percoll gradient fractionation protocol (Fig. 1A), we isolated the MAM (shown by electron micrograph in Fig. 1B) from the mitochondria, as well as from the microsomes (here in referred to as ER [37]) and cytosol [32]. During Percoll gradient fractionation, the MAM is the diffuse, white band above the heterogeneous bands that comprise mitochondria (Fig. 1C). Biochemical fractions isolated from PH5CH8 human non-neoplastic hepatocytes were subjected to immunoblot analysis using organelle-specific markers to confirm that the MAM is enriched for the known MAM-marker protein FACL4 [38–40] and is devoid of the representative mitochondrial and cytosolic proteins, Cox-1 and tubulin, respectively (Fig. 1D).

To globally define the dynamics of protein trafficking within the MAM, ER, and cytoplasm during RNA virus infection, we used Percoll gradient fractionation to isolate the MAM, ER, and cytoplasm from either PH5CH8 [30] or Huh7 human hepatocyte lines following RNA virus infection (Sendai virus) or viral RNA replication (HCV K2040 replicon system that replicates HCV RNA as a well-accepted model of chronic HCV infection [29,41,42], as well as uninfected controls, and characterized the proteome of each biochemical fraction (Fig. 1E). For these experiments, we used two different human hepatocyte cell lines (PH5CH8 and Huh7) to reduce the likelihood of characterizing cell-type specific phenomena and to focus on pathways that are generally co-regulated by RNA viruses in hepatocytes. The comparison of the proteomic changes that occur in different subcellular compartments during these two different RNA viruses (HCV and SenV) allows us to interrogate differential signaling responses during acute and chronic RNA virus infection/replication, as while both HCV and SenV activate RIG-I pathway signaling [21,22], only SenV activates signaling downstream of MAVS because the HCV NS3/4A protease cleaves MAVS during HCV replication to impair this innate immune signaling [20,24,26]. Additionally, infection with SenV can be used to model changes that occur during an acute RNA virus infection of only 8 hours, while HCV replication can model changes that occur during chronic virus replication.

**Fig 2:**
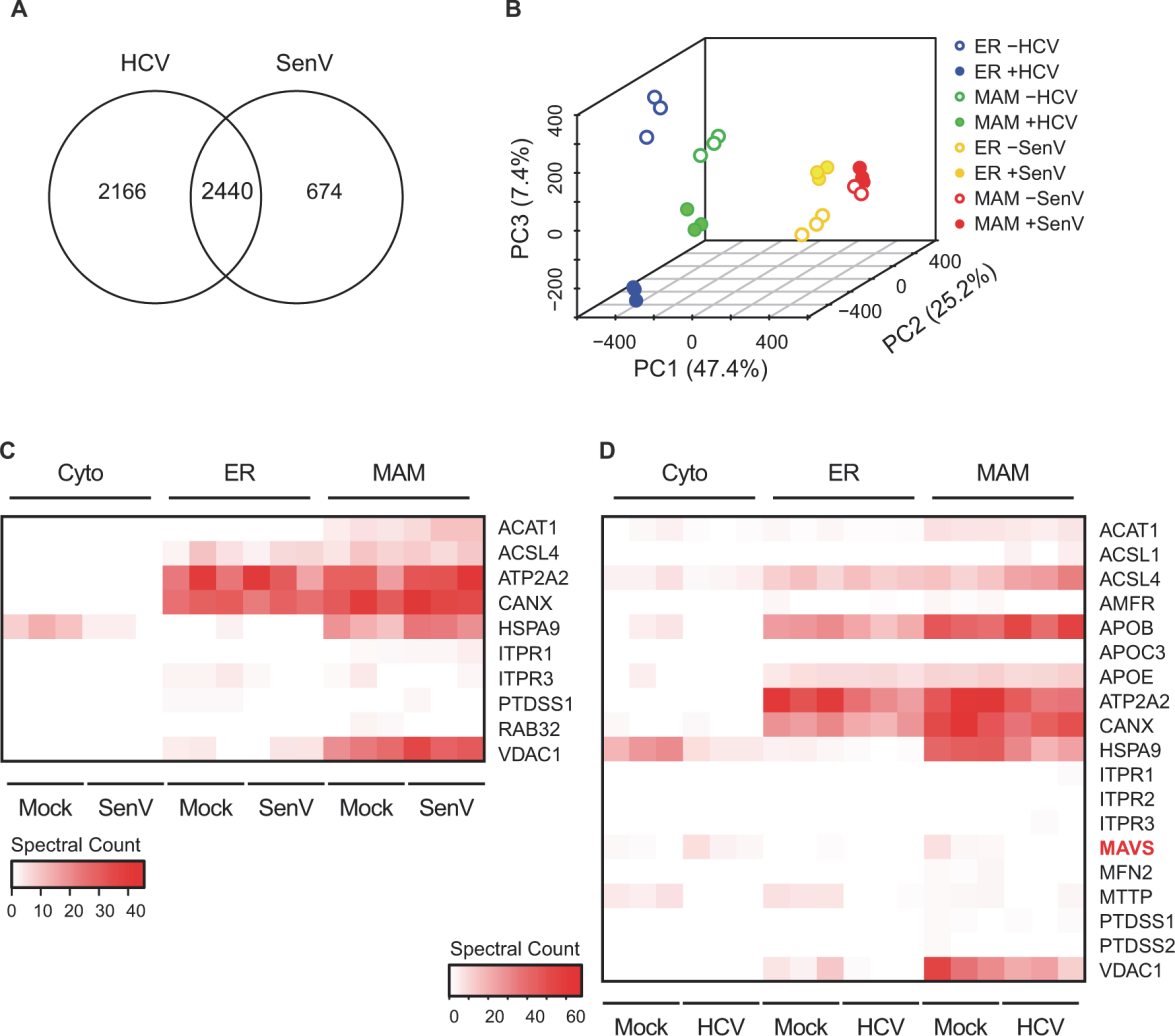
Proteomic analysis of subcellular fractions during RNA virus replication. (A) Venn diagram of total numbers of proteins identified in mock or virus samples in all biochemical fractions from Huh7 cells (mock or replicating HCV RNA) and from PH5CH8 cells (mock or after 8h of SenV infection). (B) Principal component analysis of mock and virus samples for both HCV and SenV from ER and MAM fractions. Each colored dot (open circle: no virus; closed circle; with virus) represents a technical replicate sample for each condition. The graph shows the first three independent principal components (PC1, PC2, and PC3), along with the percentage of variance captured within each component in parentheses. (C, D) Heat map of expression of proteins identified within subcellular fractions (Cytoplasm (Cyto), ER, and MAM) in SenV (C) or HCV (D) and mock samples, cross-referenced to a list of proteins known to have MAM localization (see S2 Table). The intensity of the red color indicates the normalized spectral counts of each protein across technical triplicates, as shown in the key. In (D) MAVS is highlighted in red to indicate the importance of its localization profile in HCV biology.

Following biochemical fractionation, equivalent amounts of subcellular fractions were subjected in triplicate to tandem mass spectrometry. Proteomic analysis of the mass spectrometry data identified 4606 distinct proteins in uninfected or HCV-replicating Huh7 cells, and 3114 proteins in uninfected or SenV-infected PH5CH8 cells, with a considerable overlap of total identified proteins between the two cell types (Fig. 2A; S1 Table). We used principal component analysis to compare protein expression patterns across different organelles in both viral systems (Fig. 2B). Although the MAM is a membrane subdomain of the ER [43], the two organelles are easily distinguishable by this analysis. Additionally, the presence of RNA virus (either replicating HCV RNA or infection with SenV) shifts the identified proteomics pattern into a distinct cluster, indicating differential changes in the overall protein composition of each organelle during infection. These changes may reflect both virus-driven alterations of the host cell, as well as innate immune response-mediated protein relocalization triggered by host recognition of viral infection.

To determine if the proteins we identified in the MAM reflect the known protein composition of the MAM, we cross-referenced our proteomics data set obtained from Huh7 and PH5CH8 cells to a compiled list of known MAM-localized proteins from other cell types (S2 Table) [38–40,44–58]. The results are displayed in heat maps, which display normalized protein spectral counts identified by the mass spectrometry of isolated subcellular fractions during SenV-infection and HCV replication, as compared to the list of previously characterized MAM proteins (Fig. 2C-D; see S2 Table). This analysis reveals that our biochemically-isolated MAM fractions are enriched with known MAM-localized proteins, with several localizing to both ER and MAM fractions as expected, because the MAM is contiguous with the ER. Our proteomic analysis of mock-infected Huh7 cells also identified MAVS in the MAM fraction (Fig. 2D), which we have previously shown to be localized to the MAM by immunoblotting of MAM fractions [9]. Importantly, our proteomic analysis detected a loss of MAVS from the MAM fraction in cells replicating HCV RNA and a re-localization of MAVS to the cytoplasmic fraction (Fig. 2D). This MAVS localization profile is consistent with the known biology of this system in which the HCV NS3/4A protease cleaves MAVS from the MAM during HCV RNA replication, resulting in its redistribution to the cytosol [9]. Taken together, these data indicate that Percoll gradient biochemical fractionation does indeed isolate MAM and that the subsequent proteomic analysis will detect known biological processes.

### Identification of novel MAVS-interacting proteins

We focused largely on defining the protein changes that occur on the MAM, as this is a known site for innate immune signaling [9]. To look at the comprehensive changes in protein localization on the MAM following RNA virus infection, we defined a significant shift in a protein's MAM-localization during HCV RNA replication or SenV infection as compared to mock-infected cells as at least a 2-fold increase or decrease in protein spectral counts with a Benjamini-Hochberg corrected p-value of less than 0.05. We found that several hundred proteins changed their expression in the MAM during SenV (S1A Fig.) or HCV (S1B Fig.) as compared to mock-treated cells (S3 Table). To identify groups of proteins with similar localization profiles, we used hierarchical clustering by Euclidean distance and categorized proteins from each cluster according to functional category using Gene Ontology (GO) biological processes determined by the DAVID functional annotation tool [59,60]. During SenV infection, proteins involved in respiration were upregulated in the MAM, while proteins involved in cell cycle were downregulated in the MAM (S1A Fig.). Interestingly, during HCV replication, proteins involved in respiration were downregulated in the MAM, while those involved in translational elongation and actin-based processes were recruited to the MAM (S1B Fig.). These data indicate that proteins are differentially localized to the MAM during RNA virus infection and suggests that different MAM-regulatory mechanisms exist between SenV and HCV, either due to virus-specific processes or to differential innate immune signaling between these viruses.

**Fig 3:**
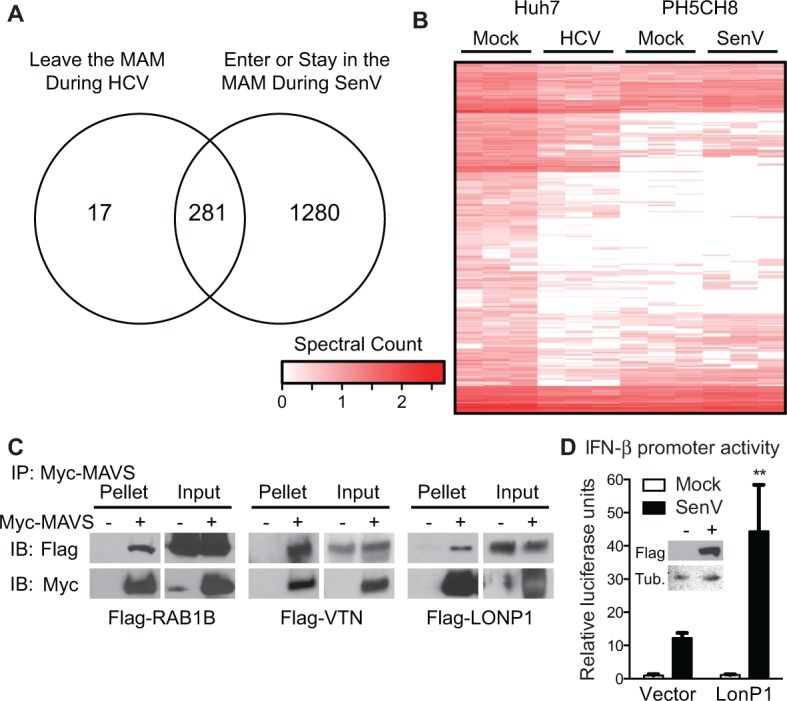
Identification of MAVS-interacting proteins using MAM proteomics. (A) Venn diagram of proteins that share a MAM-localization profile similar to that of MAVS. Proteins that move out of the MAM fraction during HCV (greater than 2-fold change over mock with a Benjamini-Hochberg corrected p-value of < 0.05) were compared to those that either move into the MAM or remain localized to the MAM during SenV infection. The 281 proteins in the intersection share this MAVS-localization pattern in the MAM during RNA virus infection. (B) Heat map of the 281 proteins with “MAVS-like” localization identified in MAM fractions from HCV and SenV, as well as mock (see panel A). Log_10_-transformed spectral counts across technical triplicates are represented by the color intensity shown in the key. (C) Immunoblot analysis of lysate (Input) and anti-Myc immunoprecipated extracts (Pellet) from cells expressing Myc-MAVS (+) or vector (-), and either Flag-RAB1B, Flag-VTN, or Flag-LONP1. (D) IFN-β promoter reporter luciferase expression of Huh7 cells expressing empty vector or Flag-LONP1 and then mock or SenV infected (24h). Values are mean -/+ SD (n = 3) of one of three replicate experiments; **P<0.01. (Inset) Immunoblot for Flag-LONP1 and tubulin (Tub.) protein expression.

We sought to employ the data from this proteomic analysis to identify novel regulators of the MAVS-signaling pathway, which we have previously shown can assemble into signaling-protein complexes in the MAM [9]. In particular, we hypothesized that proteins with the same localization pattern as MAVS in the RNA-virus infected cell would be potential MAVS-interacting proteins. The MAVS-localization pattern is characterized as leaving the MAM during HCV replication and either being in present or re-localized to the MAM during SenV. To determine the proteins that have a similar localization profile with MAVS in the RNA virus-infected cell, we classified our proteomics data according to these possible MAVS-localization profiles in both HCV and SenV. We found 281 proteins that share the MAVS localization pattern (Fig. 3A). The relative levels of these 281 candidate MAVS-interacting proteins in the MAM during RNA virus or mock-treatment is displayed in Fig. 3B. We tested three of these proteins (RAB1B, Vitronectin (VTN), and Lon Peptidase 1(LONP1)) for interaction with MAVS. In co-immunoprecipitation assays in Huh7 cells using over-expressed epitope tagged proteins, an interaction was detected with Myc-tagged MAVS and Flag-RAB1B, Flag-VTN, and Flag-LONP1 (Fig. 3C). We next determined if one of our identified MAVS-interacting proteins, LONP1, regulated the RIG-I/MAVS-signaling pathway by measuring SenV-mediated signaling to the IFN-β promoter in promoter luciferase assays following over-expression of LONP1. While over-expression of LONP1 on its own did not drive signaling to the IFN-β promoter, LONP1 over-expression during SenV resulted in augmented SenV-signaling to IFN-β (Fig. 3D). Therefore, by identifying proteins with localization patterns similar to MAVS in the MAM during RNA virus infection, we have found 3 novel MAVS-interacting proteins and demonstrated that one of these MAVS-interacting proteins, LONP1, augments RIG-I pathway signaling. It is possible that some of the remaining 278 proteins with MAVS-localization profiles are also MAVS-interacting and innate immune regulatory proteins.

### Protein networks enriched on the MAM during RNA virus infection

**Fig 4:**
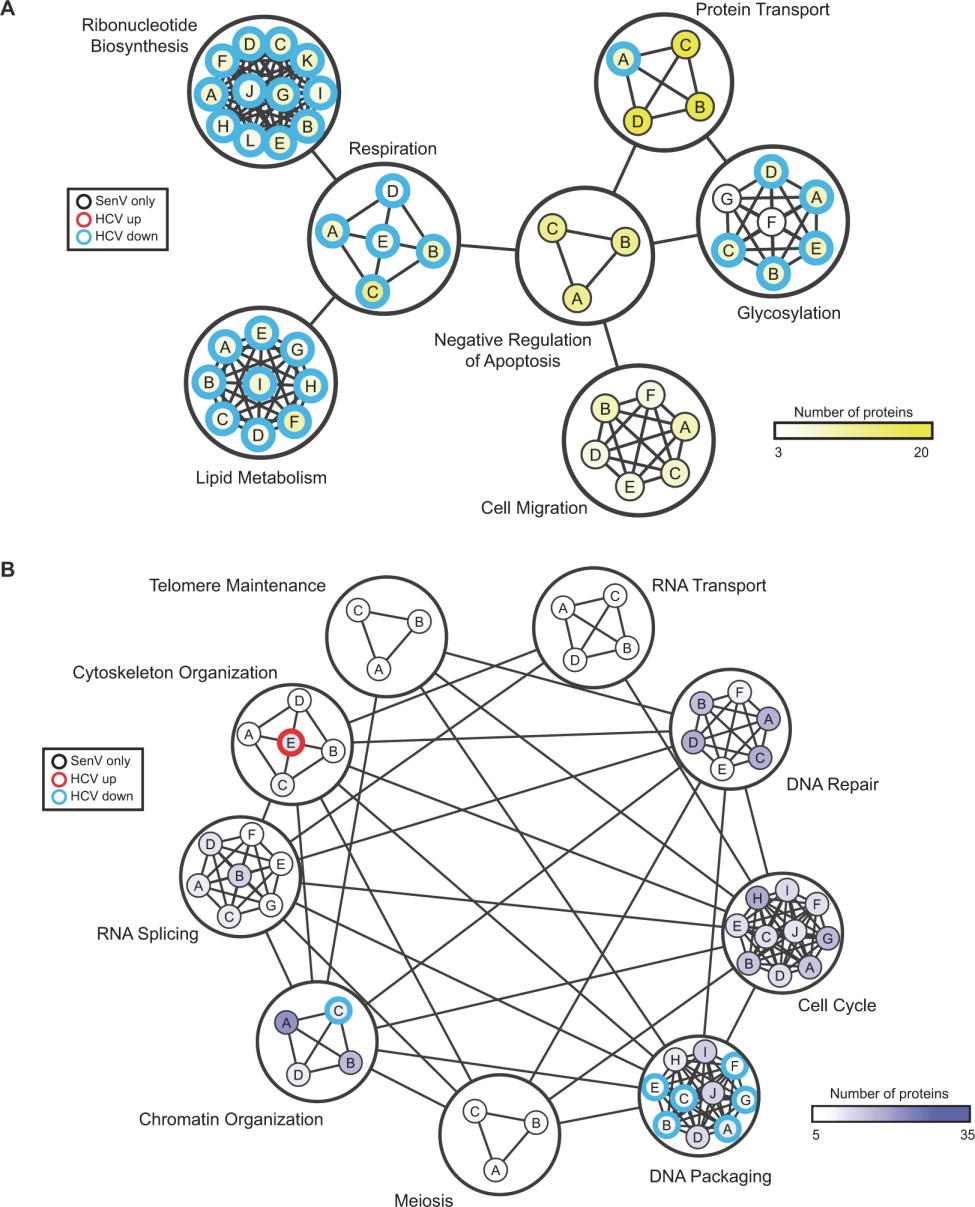
Network enrichment map of protein dynamics on the MAM during RNA virus replication. Enrichment map for proteins moving into the MAM with SenV (A) or leaving the MAM with SenV (B), compared to the proteins identified on the MAM during HCV. Nodes represent enriched biological pathways grouped by DAVID-identified clusters, manually circled and classified by function, for proteins differentially expressed in the MAM fraction following SenV (≥2-fold; BH-adjusted p-value < 0.05). Nodes were classified by Functional Annotation Clustering of Gene Ontology (GO) biological pathways using the DAVID bioinformatics resource. Edges between nodes represent overlap between two pathways, and edges between functional category clusters represent at least one shared protein. Node internal color is proportional to the number of proteins comprising the canonical pathway (darker shading in the node means greater number of proteins). Node edges in red represent pathways enriched in the MAM in cells replicating HCV and SenV, and node edges in blue represent pathways enriched in groups of proteins leaving the MAM in cells replicating HCV and SenV. Nodes edges in black represent SenV-specific changes. Node identity is depicted by the letter within each node, and the key is listed in S4 Table.

To identify functional biological networks of proteins that had dynamic localization to or away from the MAM during RNA virus infection, we used enrichment map analysis of GO biological processes. Surprisingly, we did not identify any categories enriched with proteins moving into the MAM during both SenV and HCV (Fig. 4A), although we did find that there are five proteins that move into the MAM during both SenV and HCV (S2A Fig.).

On the other hand, during infection with SenV, proteins mapping to GO biological processes such as cell migration, negative regulation of apoptosis, and protein transport were all upregulated in the MAM (Fig. 4A; Table 1 and S4 Table). Interestingly, several categories were regulated in opposite fashion during SenV and HCV (move into the MAM during SenV and leave the MAM during HCV; a similar localization pattern as MAVS). These include ribonucleotide biosynthesis, glycosylation, respiration, and lipid metabolism (Fig. 4A). An analysis of the top regulated GO biological processes for proteins entering the MAM during SenV and HCV reveals the different biological processes that are recruited to the MAM by these two RNA viruses. For SenV, these include the processes of lipid and fatty acid oxidation, while for HCV these processes include actin/cytoskeleton organization (Table 1 and S4 Table).

**Table 1:** Gene Ontology Analysis for proteins that enter the MAM during RNA virus.

Biological Process					
SenV	N	p-value	HCV	N	p-value
GO:0055114∼Oxidation reduction	28	9.1E-04	GO:0006414∼Translational elongation	25	1.2E-17
GO:0016044∼Membrane organization	20	4.7E-03	GO:0006412∼Translation	32	3.7E-11
GO:0006091∼Generation of precursor metabolites and energy	18	5.2E-03	GO:0030029∼Actin filament-based process	20	8.5E-05
GO:0015980∼Energy derivation by oxidation of organic compounds	12	1.3E-02	GO:0007010∼Cytoskeleton organization	26	3.5E-04
GO:0018196∼Peptidyl-asparagine modification	5	1.4E-02	GO:0030036∼Actin cytoskeleton organization	17	4.2E-03
**Cellular Component**					
**SenV**	**N**	**p-value**	**HCV**	**N**	**p-value**
GO:0005739∼Mitochondrion	60	1.6E-14	GO:0005829∼Cytosol	80	1.0E-19
GO:0044429∼Mitochondrial part	41	9.1E-12	GO:0005856∼Cytoskeleton	74	6.1E-15
GO:0031090∼Organelle membrane	56	1.0E-11	GO:0043228∼Non-membrane-bounded organelle	105	1.6E-14
GO:0042470∼Melanosome	16	1.3E-08	GO:0043232∼Intracellular non-membrane-bounded organelle	105	1.6E-14
GO:0048770∼Pigment granule	16	1.3e-08	GO:0031090∼Organelle membrane	100	4.7E-33

Shown are the top 5 pathways that are enriched under each condition, the number of proteins that fall within each category (N), and the p-values obtained.

Interestingly, the top GO cellular components recruited to the MAM during SenV all have to do with mitochondrial features and metabolism (Table 1), suggesting that MAM and mitochondria might form close interactions during RIG-I pathway activation upon SenV infection and supports the idea of innate immune synapses forming between the membranes of the ER and mitochondria at the MAM during RIG-I pathway activation [9]. The top GO cellular components recruited to the MAM during HCV are different than those during SenV, and include actin cytoskeleton and non-membrane-bounded organelles (Table 1).

Enrichment map analysis of GO biological processes that move out of the MAM during SenV infection reveals an overwhelming bias toward nuclear components (Fig. 4B; Table 2 and S4 Table). While HCV-infected MAM fractions do demonstrate slight enrichment toward nuclear pathways in leaving the MAM, the primary biological processes and cellular components moving out of the MAM during HCV infection map to mitochondrial features (Fig. 4B; Table 2 and S4 Table). We also found that there are 17 proteins in common that are lower in expression in MAM during SenV and HCV (S2B Fig.). Taken together, this network analysis shows that during SenV, mitochondrial components move into the MAM, while nuclear components leave the MAM; however, during HCV, mitochondrial components leave the MAM while actin-related processes are moving into the MAM. This analysis suggests that during RNA virus infection there is a reorganization of intracellular organelles, perhaps related to differential regulation of innate immune signaling pathways by SenV and HCV.

**Table 2:** Gene Ontology Analysis for proteins that leave the MAM during RNA virus.

Biological Process					
SenV	N	p-value	HCV	N	p-value
GO:0051276∼Chromosome organization	35	3.0E-10	GO:0055114∼Oxidation reduction	53	5.3E-16
GO:0006396∼RNA processing	34	4.3E-08	GO:0006091∼Generation of precursor metabolites and energy	34	2.4E-12
GO:0006414∼Translational elongation	16	8.2E-08	GO:0045333∼Cellular respiration	19	5.6E-10
GO:0006397∼mRNA processing	24	2.2E-06	GO:0006119∼Oxidative phosphorylation	19	6.7E-10
GO:0006333∼Chromatin assembly or disassembly	16	2.3E-06	GO:0015980∼Energy derivation by oxidation of organic compounds	20	6.5E-8
**Cellular Component**					
**SenV**	**N**	**p-value**	**HCV**	**N**	**p-value**
GO:0043228∼Non-membrane-bounded organelle	119	5.8E-25	GO:0005739∼Mitochondrion	128	2.7E-58
GO:0043232∼Intracellular non-membrane-bounded organelle	119	5.8E-25	GO:0044429∼Mitochondrial part	97	2.3E-54
GO:0005694∼Chromosome	45	7.6E-18	GO:0031980∼Mitochondrial lumen	53	3.8E-35
GO:0044427∼Chromosomal part	36	7.7E-13	GO:0005759∼Mitochondrial matrix	53	3.8E-35
GO:0043233∼Organelle lumen	76	1.1E-10	GO:0031090∼Organelle membrane	100	4.7E-33

Shown are the top 5 pathways that are enriched under each condition, the number of proteins that fall within each category (N), and the p-values obtained.

### Proteins co-regulated on the MAM during RNA virus replication

**Fig 5:**
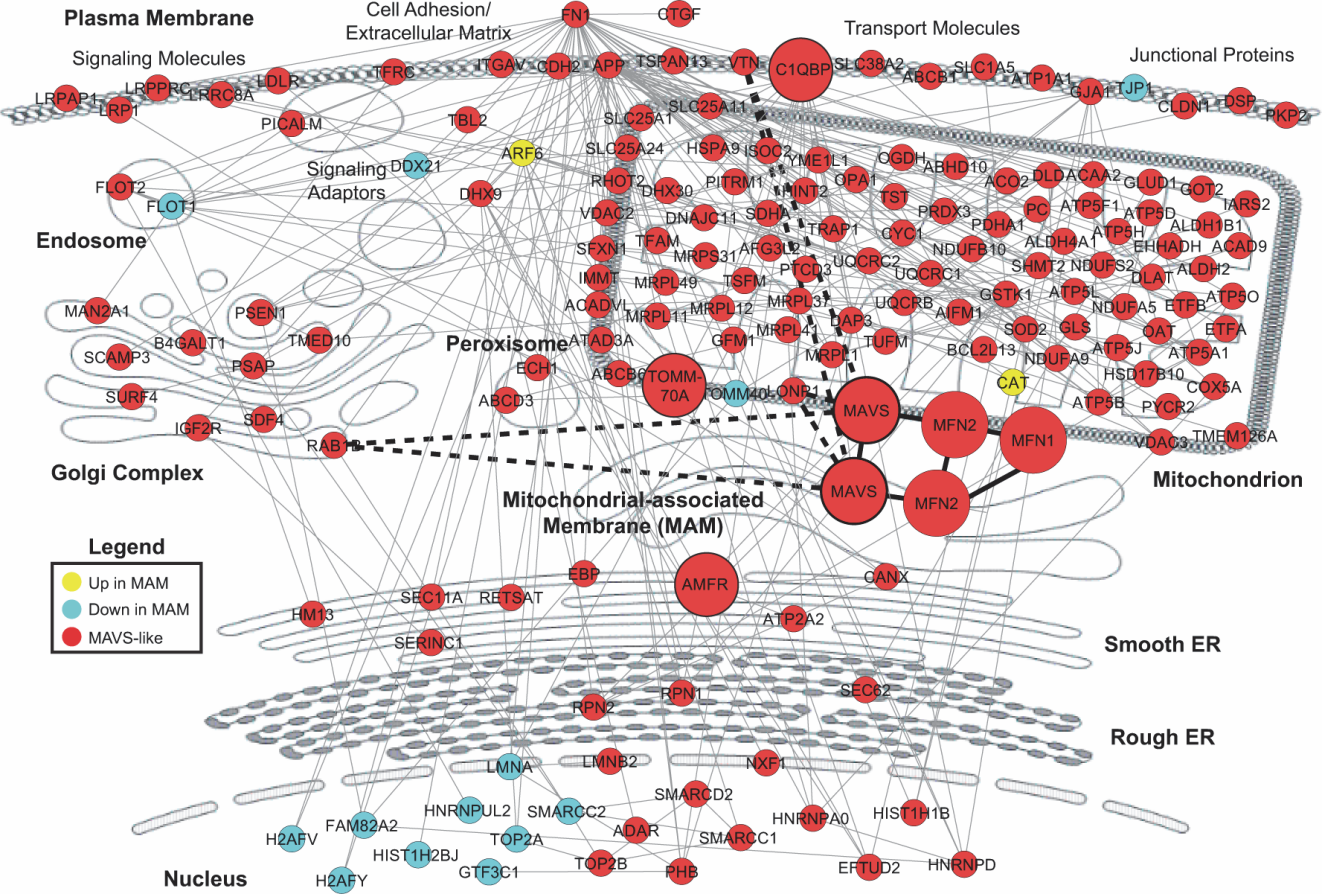
Cellular interaction network of proteins identified with dynamic MAM localization during RNA virus replication. Cellular mapping of the interaction network of proteins identified in MAM fractions that either increase in the MAM with HCV and SenV (yellow), decrease in the MAM with both HCV and SenV (blue), or that have “MAVS-like” localization (red; decreased in the MAM with HCV, increased or unchanged in MAM with SenV). The interaction network was determined by using InnateDb and visualized with Cytoscape. It includes all proteins that interact with at least one other protein. Bold-faced dotted edges represent protein-protein interactions discovered and validated in this study. Previously described MAVS-interacting proteins (MAVS, MFN1, MFN2, C1QBP, AMFR, and TOMM70) are highlighted by large red circles. Nodes representing each protein were positioned on a cell map according to subcellular localization using annotation information from InnateDb.

To better understand the protein complexes that change in the MAM during RNA virus infection, we classified proteins on the MAM according to their localization changes: (1) those that enter the MAM during both HCV and SenV, (2) those that leave the MAM during both viruses, and (3) those with a MAVS-like localization in which they enter the MAM fraction during SenV infection and leave during HCV. All the proteins identified by mass spectrometry falling into one of these three categories were assessed for interactions among each other using the InnateDb interface [61]. Proteins that share a physical interaction with at least one other in this list were mapped according to their defined subcellular localization curated using InnateDb, and the network was plotted in Cytoscape [62], with nodes manually adjusted to overlay a diagram of the cell (Fig. 5). We found that many proteins typically localized to non-MAM cellular compartments interact with each other in the MAM during RNA virus replication, as visualized by the many identified edges between the nodes. This mapping suggests a dynamic interplay among subcellular membranes allowing for protein complex organization during RNA virus infection.

## Discussion

In this study, we analyzed the proteome of the MAM during two different RNA virus infections to identify proteins that were targeted to or away from the MAM during the antiviral response. Our underlying hypothesis was that protein regulators of antiviral signaling would change their expression on the MAM upon induction of antiviral signaling. Our results demonstrate that there are many proteins changing in expression on the MAM during RNA virus infection. Many of these proteins are differentially regulated during SenV and HCV but are found to be in a physical interaction network, suggesting that these proteins are co-regulated during the antiviral response.

Proteomic analysis of the MAM during RNA virus infection allows us to capture the global dynamics of protein changes occurring on the MAM during RNA virus infection. Our results indicate that our MAM isolation protocol is indeed purifying the MAM, as the proteomics of this biochemical fraction identified many proteins that have previously been reported to have MAM localization. In this study, we performed proteomic analysis of the MAM, ER, and cytoplasm. As MAVS localizes to MAM, mitochondria, and peroxisomes[9,13,14], future studies including proteomic analysis of pure mitochondria with no contaminating MAM as compared to the MAM during RNA virus infection would provide insight into how the antiviral response is differentially regulated from the MAM or mitochondria. While proteomic analysis of peroxisomes would also be of interest, to date we have been unable to purify peroxisomes devoid of MAM contamination (Horner and Gale, unpublished results).

In our study, we found many proteins differentially regulated in the MAM during SenV and HCV. These proteins share a similar localization profile with MAVS, a portion of which is localized to the MAM, but then upon on HCV NS3/4A-cleavage of MAVS, are no longer present in the MAM. We tested three of those proteins (out of a possible 278) for interaction with MAVS and found that all three interacted with MAVS in co-immunoprecipitation experiments. Therefore, by knowing the localization of a protein and how it changes during RNA virus infection and comparing this localization with other proteins that share this localization pattern, we can predict new interacting proteins. Here, we described three new MAVS-interacting proteins (RAB1B, VTN, LONP1) and defined LONP1 was a positive regulator of the RIG-I/MAVS signaling pathway. While we have yet to determine if RAB1B and/or VTN are MAVS-regulatory proteins, literature assessment of each suggests possible roles in MAVS signaling regulation in the innate immune response to RNA viruses. RAB1B is a GTPase involved in vesicle transport between the ER and the Golg i[63]. ER-Golgi transport has previously been implicated in signaling between MAVS and TRAF3, an innate immune signaling protein downstream of MAVS [64], suggesting that RAB1B could be a chaperone protein regulating signaling between MAVS and TRAF3, and that this signaling would be disrupted by the HCV NS3/4A protease. While VTN, a glycoprotein known to be involved in cell adhesion and localized to focal adhesions [65], has not been implicated in RIG-I/MAVS signaling before, both the focal adhesion kinase and the actin cytoskeleton are required for RIG-I/MAVS signaling [66,67]. Therefore, VTN may be involved in providing cytoskeletal support for RIG-I signaling. In our proteomic analysis, we also found the MAM-associated protein AMFR/GP78 has the same localization pattern as MAVS, and AMFR/GP78 was recently described as a regulatory protein of MAVS and the innate immune response [68]. Taken together, these data support a role for MAM-associated proteins that interact with MAVS upon RIG-I pathway activation as regulators of the antiviral response.

While we did identify the ATP-dependent serine protease LONP1 as interacting with MAVS and functioning as positive regulator of MAVS-signaling in the context of SenV infection, the mechanism of this innate immune regulatory role has yet to be defined. LONP1 is required for mitochondrial protein quality control and mitochondrial biogenesis and function, as well as having some chaperone activity [69,70]. While synthesized on the rough ER, LONP1 is localized to the mitochondrial matrix. How and why is this mitochondrial matrix localized protein localized to the MAM following SenV? In fact, our proteomic analysis reveals that many mitochondrial proteins are detected in the MAM fraction following SenV (but not with HCV), as shown in Fig. 5. Changes in expression of individual proteins detected by MAM proteomics may actually reflect changes in organelle interactions during the antiviral signaling response and the generation of new signaling sites through these organelle interactions. In support of this idea, mitochondria are known to elongate during RIG-I pathway activation [71], and our previous imaging analysis reveals that MAM and mitochondria, along with peroxisomes, do form extensive contacts during RIG-I pathway activation [9]. The ability to fractionate the MAM from mitochondria may be compromised during antiviral signaling in such a way that during RIG-I pathway activation MAM and mitochondria co-fractionate, likely due to mitochondrial elongation. Therefore, we conclude that activation of the innate immune response alters organelle interactions. The reason for altered organelle interactions during the innate immune response is unknown, but they could be important for the energetic and metabolic requirements of antiviral signaling, all housed within different organelles and effectively shared during signaling activation in membrane signaling microdomains [72], such as those provided by the MAM at the contact sites between the ER and mitochondria. Indeed, lipid signaling microdomains are specialized centers in the cell for rapid communication and information transfer [73] that are likely important for a properly regulated antiviral response.

This study represents the third time the MAM proteome has been described [74,75], but the first time that it has been described in the context of RNA virus infection and innate immunity. Previously, the MAM proteome has been characterized from mouse brain samples and also from human fibroblasts during human cytomegalovirus (HCMV) infection [74,75]. The HCMV glycoprotein pUL37x1 targets ER-mitochondrial contacts at the MAM to regulate apoptosis during infection [76], and proteomic analysis of the MAM during HCMV infection revealed that, similar to our findings with SenV, many mitochondrial proteins were re-localized into the MAM [75]. As HCMV is a DNA virus and SenV is an RNA virus, the mechanisms used by these viruses for altering membrane interactions are likely different, and it remains to be determined whether changes in membrane interactions are beneficial to the virus (as is likely the case for HCMV) or not. Interestingly, HCV does not induce increases in mitochondrial/MAM interactions during the viral replication cycle, even though it is known to induce membrane rearrangements during replication and targets the MAM through both its NS3/4A and Core proteins [9,77–79]. Perhaps these reduced MAM/mitochondrial associations during HCV are due to NS3/4A cleavage of MAVS preventing a MAVS-oligomerization event that is required for MAM/mitochondrial interactions. Alternatively, our inability to detect mitochondria in the MAM fraction during HCV replication could very likely be a result of depletion of mitochondria by mitophagy during HCV replication [80]. Whether this mitophagic event is an active event on the part of HCV to disrupt innate immune signaling at the MAM-mitochondrial interface remains to be determined.

Overall, our proteomic analysis of subcellular fractions during RNA virus infection reveals dramatic alterations in cell biology and highlights the MAM as a key platform for housing the dynamic protein re-localizations that occur during RNA virus infection. As the MAM has been implicated in innate immunity, inflammation, and neurological diseases such as Alzheimer’s, continued analysis of the MAM and MAM-regulated processes, will be important to understand how the MAM contributes to these different processes and to developing strategies designed to target the MAM to prevent disease.

## Supporting Information

S1 Fig Differential protein localization to the MAM following virus infection. Heat map of protein expression in the MAM representing changes of at least 2 fold following virus infection with SenV (A) or during HCV replication (B), as compared to mock. Proteins are ordered by hierarchical clustering, and proteins with similar patterns based on hierarchical clustering were analyzed for GO biological process by DAVID and labeled with most common process identified. (BH-adjusted p-value < 0.05; log_10_ scale based on spectral count; technical triplicates plotted).
(EPS)

S2 Fig Venn diagram analysis of proteins co-regulated in the MAM during RNA virus. (A) Venn diagram of proteins whose expression is higher in the MAM during HCV or SenV as compared to mock. (B) Venn diagram of proteins whose expression is lower in the MAM during HCV or SenV as compared to mock.
(EPS)

S1 Table Peptide-level spectral counts for proteomics data. Proteins are labeled by their International Protein Index (IPI) identification, along with the official protein name and an official description of the protein. The identified peptide is described by its sequence position in the protein, along with the identified peptide itself. Spectral counts identified across each sample are listed. The tabs on the Excel spreadsheets provide access to the Huh-based HCV data and the PH5CH8-based SenV data.
(XLSX)

S2 Table List of known MAM-localized proteins. This table lists proteins previously referenced as being localized to the MAM, including those found in the following references [81–89].
(DOCX)

S3 Table List of proteins identified by proteomics analysis that are upregulated or downregulated in the MAM during RNA virus infection. (PDF)

S4 Table DAVID functional annotation clustering of proteins entering the MAM or leaving the MAM with SenV. (XLSX)
