# Correction: Proteomic Analysis of Mitochondrial-Associated ER Membranes (MAM) during RNA Virus Infection Reveals Dynamic Changes in Protein and Organelle Trafficking

**DOI:** 10.1371/journal.pone.0124226

**Published:** 2015-04-13

**Authors:** The PLOS ONE Staff

The affiliation for the first author is incorrect. Stacy M. Horner is affiliated with 1 Department of Immunology, University of Washington, Seattle, Washington, United States of America, 2 Department of Molecular Genetics and Microbiology, Duke University Medical Center, Durham, North Carolina, United States of America, and 3 Department of Medicine, Duke University Medical Center, Durham, North Carolina, United States of America

## References

[1] Horner SM, Wilkins C, Badil S, Iskarpatyoti J, Gale M Jr (2015) Proteomic Analysis of Mitochondrial-Associated ER Membranes (MAM) during RNA Virus Infection Reveals Dynamic Changes in Protein and Organelle Trafficking. PLoS ONE 10(3): e0117963 doi:10.1371/journal.pone.0117963 25734423 10.1371/journal.pone.0117963 PMC4348417

