# Supplementary material for: Proteomic Analysis of Mitochondrial-Associated ER Membranes (MAM) during RNA Virus Infection Reveals Dynamic Changes in Protein and Organelle Trafficking
Source: PLoS One. 2015 Mar 3;10(3):e0117963. doi: 10.1371/journal.pone.0117963 (PMC4348417; doi:10.1371/journal.pone.0117963)
Supplement: S2 Table — This table lists proteins previously referenced as being localized to the MAM, including those found in the following references [81–89]. (DOCX) [file pone.0117963.s004.docx]

Table S2. List of known MAM-localized proteins

| **MAM-localized proteins** | **Reference** |
| --- | --- |
| HSPA9 (GRP75) | [81] |
| ACAT1 (SOAT1) | [48,55,58,82] |
| ITPR1 | [47,81,83,84] |
| ITPR2 | [44] |
| ITPR3 (IP3R3) | [45-47,52] |
| HSPA5 (GRP78; BIP) | [55] |
| ACSL1 | [38,39] |
| ACSL4 (FACL4) | [38-40] |
| CANX | [58] |
| PDIA3 (ERp57) | [57] |
| PDIA10 (ERp44) | [54-56] |
| ATP2A2 (SERCA2b) | [44,52,53] |
| PTDSS1 (PSS1) | [51] |
| PTDSS2 (PSS2) | [51] |
| MFN2 | [50] |
| DGAT2 | [48,49] |
| SIGMAR1 (OPRS1) | [83] |
| VDAC1 | [81] |
| AMFR (RNF45; GP78) | [85] |
| ERO1-alpha | [54,55] |
| PS2 | [86] |
| MTTP | [48,82] |
| SCD1 | [82] |
| PEMT | [87] |
| ApoB | [88] |
| ApoC | [88] |
| ApoE | [88] |
| PML (extranuclear) | [46] |
| PACS2 | [40] |
| RAB32 | [89] |
