# Supplementary material for: Proteomic Analysis of Mitochondrial-Associated ER Membranes (MAM) during RNA Virus Infection Reveals Dynamic Changes in Protein and Organelle Trafficking
Source: PLoS One. 2015 Mar 3;10(3):e0117963. doi: 10.1371/journal.pone.0117963 (PMC4348417; doi:10.1371/journal.pone.0117963)
Supplement: S3 Table — (PDF) [file pone.0117963.s005.pdf]

HCV		SenV	
**Enter MAM**	**Leave MAM**	**Enter MAM**	**Leave MAM**
A2M	ABCB1	ABCC1	AAAS
AARS	ABCB6	ABCD3	ABCE1
ABCE1	ABCD3	ACADVL	ACTR2
ACBD5	ABHD10	ACAT1	AKAP2
ACSL1	ACAA2	ACO2	ALPP
ACSL4	ACAD9	ACOX1	ANLN
ACTN1	ACADM	ACSL3	AP2A1
ACTN4	ACADSB	ADAM9	AP2B1
ADD1	ACADVL	AGPS	APEX1
ADFP	ACO2	AGR2	APOBEC3B
AGT	ADAM9	AGTRAP	ARHGAP17
ALDH1A1	ADAR	ALDH18A1	ATAD3C
APEX1	AFG3L2	ALDH1B1	ATP2B4
ARF6	AFP	ALG1	ATP6AP1
ARHGAP5	AGK	ANPEP	BAIAP2
ASAH1	AGMAT	ANXA1	BAZ1A
ATP2B4	AGRN	ANXA4	BAZ1B
ATXN2	AHSG	ANXA6	BUD31
BAIAP2L1	AIFM1	APOL2	C20orf20
BCAM	AK2	ARF6	C8orf55
BIN1	AK3L1	ATP1A1	CHD4
CALD1	ALDH16A1	ATP1B1	CHD7
CALU	ALDH18A1	ATP2B1	CHST12
CAP1	ALDH1B1	ATP6V1B1	CKAP5
CAT	ALDH2	B4GALT5	CSNK1A1
CD38	ALDH4A1	BST2	CSRP1
CDC42EP4	AMFR	C11orf59	CSTF3
CDK5RAP3	AMT	C14orf156	CUX1
CEACAM1	ANPEP	C1QBP	DDX21
CFL1	APP	C3orf21	DEK
CHD4	ATAD3A	C4B	DHRS7B
CKAP5	ATP1A1	CAT	DNAJB12
COBLL1	ATP2A2	CD109	DNAJC3
COL18A1	ATP5A1	CD276	DNMT1
CORO1B	ATP5B	CDCP1	DOCK5
CPVL	ATP5D	CHID1	DPY30
CRELD1	ATP5F1	CKAP4	DYNC1H1
CRELD2	ATP5H	CLPTM1L	DYNC1I2
CSRP1	ATP5J		DYNC1LI2
CTNNAL1	ATP5L		DYNLT1
CTSB	ATP5O		EDF1

HCV		SenV	
Enter MAM	Leave MAM	Enter MAM	Leave MAM
CYP51A1	ATP6V0A1	CNP	EEF1A1
DGAT1	B3GNT1	CORO1A	EEF1B2
DMD	B4GALT1	COX5A	EEF1D
DNAJC1	BCL2L13	COX7A2	EEF1G
DNAJC2	C14orf156	CPNE2	EIF3F
DNM2	C1QBP	CPNE3	EIF4A3
DPM1	C5orf33	CPT1A	ELAVL1
DPP3	CANX	CTSD	EPN2
DTNA	CCDC109A	CYBRD1	ERCC6L
DYNC1H1	CCPG1	DAD1	EXOC2
DYNC1I2	CDH2	DAP3	EXOC3
DYNC1LI1	CHST13	DCBLD2	EZR
DYNC1LI2	CLDN1	DDOST	FAM82A2
DYNLT1	CLGN	DNAJB11	FBL
EEF1A1	COL5A2	DNAJC9	FLOT1
EEF1B2	COX5A	DPM1	FUBP1
EEF1D	COX6B1	DSG2	FUBP3
EEF1G	CPD	DYSF	G3BP1
EEF2	CPT2	ECHS1	GANAB
EIF5A	CTAGE5	EDIL3	GLYR1
EML4	CTGF	EEA1	GNA12
ENAH	CYB5R1	F3	GNB2L1
EPB41	CYC1	FAM3C	GTF2I
EPB41L5	DAGLB	FAR1	GTF3C1
EPCAM	DAP3	FH	H2AFV
EPS8L2	DDX21	FKBP8	H2AFX
ERLIN1	DHCR24	FLNB	H2AFY
ERLIN2	DHX30	FNDC3B	HIP1
ERO1L	DHX9	GBA	HIST1H2BJ
ESD	DLAT	GCS1	HIST1H3C
EZR	DLD	GNA13	HIST1H4H
F2	DNAJC10	GNAI1	HIST2H3A
FADS1	DNAJC11	GNB2	HMGA2
FARP1	DNAJC13	GNB4	HMGB1
FASN	DSP	GNG12	HMGB2
FERMT2	EBP	GNPAT	HMGB3
FGB	ECE1	GOLGA5	HMGN1
FGG	ECH1	GPSN2	HMHA1
FKBP11	EFTUD2	GRN	HNRNPK
GAPDH	EHHADH	GRPEL1	HNRNPM
GC	ELOVL1	HADHA	HNRNPUL2

HCV		SenV	
**Enter MAM**	**Leave MAM**	**Enter MAM**	**Leave MAM**
GCN1L1	ENPP1	HCCS	HNRPLL
GNAI2	ERMP1	SRP9	HSPA14
GNAL	ETFA	HIBADH	IFITM3
GNAQ	ETFB	HLA-B	KANK2
GNAS	FAM38A	HMOX1	KHSRP
GNB2L1	FAM62A	HMOX2	KIAA0415
GOLM1	FAM82A2	HS2ST1	KIF11
GPI	FGFR4	HSD17B4	KIF18B
H6PD	FLOT1	HSPA13	KIF4A
HCV-RNA-Pol	FLOT2	HSPA9	KTELC1
HDLBP	FLVCR1	HSPD1	LGALS3
HMGB3	FN1	ICAM1	LIMS1
HMGCS1	FOXRED1	IER3IP1	LMNA
HMHA1	GALNT2	IGF2R	LMNB1
HN1L	GFM1	ITGA5	LOC100131294
HSD17B2	GJA1	ITGB4	LOC649330
HSP90AA1	GLDC	ITM2C	LRRC8E
HSP90AB1	GLG1	ITPR1	MAP1S
HSP90B1	GLS	JAGN1	MBD3
HSPA5	GLTPD2	JUP	MDC1
HYOU1	GLUD1	KIAA0090	MGMT
IQGAP1	GOLIM4	KIAA0776	MKI67
IQGAP2	GOT2	KIAA1949	MPRIP
ITIH2	GPD2	KIRREL	MSH6
KANK4	GRHPR	KRTCAP2	MSN
KIAA0776	GRPEL1	LDLR	MYADM
KIAA1715	GSTK1	LGALS3BP	MYBBP1A
KIF21A	GTF3C1	LGALS8	MYH10
KRT1	H2AFV	LGMN	MYO1C
KRT10	H2AFY	MKKS	MYO5A
KRT2	HEATR1	LOC727947	MYST2
LEPRE1	HINT2	LONP1	NCAPD3
LMAN1	HIST1H1B	LRRC59	NDUFA10
LMO7	HIST1H1E	MDH2	NDUFS8
MARCKSL1	HIST1H2BJ	MEST	NHP2L1
MEGF9	HIST1H4H	MGAT4B	NOC2L
MTHFD1	HIST2H2AA4	MOXD1	NOLC1
MTPN	HM13	MPP6	NUMA1
MYH10	HMGCL	MRC2	NUP210
MYH14	HNF4A		NUP214
MYO1D			

HCV		SenV	
**Enter MAM**	**Leave MAM**	**Enter MAM**	**Leave MAM**
NARS	HNRNPA0	MRPL10	ORC3L
NFXL1	HNRNPC	MRPS30	PARP1
NS3	HNRNPD	NAP1L1	PARVA
PARP1	HNRNPUL2	NCAM2	PCBP2
PDE8A	HSD17B10	NDUFS1	PDIA6
PDIA3	HSPA9	NDUFS3	PDS5A
PDIA4	HSPD1	NDUFV2	PIPSL
PDIA6	HSPE1	NFKB2	PLCB3
PDLIM1	IARS2	NRP2	POFUT1
PDLIM5	IDH2	NUP62	PPAP2C
PDS5A	IDH3A	OAS1	PPP2R1A
PEBP1	IDH3B	OAT	PRKACA
PECAM1	IGF2R	OSBPL8	PRKDC
PHIP	IKIP	P4HA1	PRPF8
PHYH	IL1RAP	PARP14	PSIP1
PLCB1	IMMT	PBXIP1	PSPC1
PLS3	ISOC2	PDP1	PTBP1
PLXND1	ITGA1	PECI	PTRF
PPFIA1	ITGAV	PEX11B	PXDN
PPP1CB	ITM2B	PEX14	RAB10
PRDX1	KIAA0564	PGRMC2	RAB32
PRDX4	KIAA2013	PHB2	RAB5B
PRKAA1	LACTB	PI4K2A	RAD50
PROS1	LDLR	PLAUR	RAI14
QDPR	LETM1	PLOD1	RBM14
RCC2	LMNA	PLSCR1	RBM3
RDX	LMNB2	PPIF	RCC1
RECQL	LONP1	PPL	RCC2
RPL13A	LPHN2	PRDX3	RECQL
RPL26	LRP1	PSAP	RFC1
RPS16	LRPAP1	PSEN1	RFC4
RPS17	LRPPRC	PTGFRN	RFC5
RPS2	LRRC8A	PTRH2	RHOT1
RPS3	MAN2A1	PTTG1IP	RIF1
RPS6KA3	MAVS	RAB18	RPL39
SDF2L1	MCCC1	RAB1B	RPS10
SEC31A	MCCC2	RAB3D	RPS12
SEP11	MDH2	RAB5C	RPS16
SEP7	ME2	RHOC	RSPRY1
SEP9	MFN1	RPL10A	SAFB
SLC27A2	MFN2	RPL36	SCRIB

HCV		SenV	
**Enter MAM**	**Leave MAM**	**Enter MAM**	**Leave MAM**
SLC9A3R1	MGAT1	S100A10	SEP9
SMPDL3B	MKI67	S100A13	SFPQ
SNTB1	MLEC	SACM1L	SH3BP4
SORD	MMAB	SCARB1	SLC12A9
SPTAN1	MRPL1	SCP2	SLC19A1
SRGAP2	MRPL11	SDC4	SLC1A4
SRPR	MRPL12	SEC63	SLC27A3
STIM1	MRPL37	SERPINH1	SLC39A14
SUMO2	MRPL41	SLC12A7	SMARCA4
TLN1	MRPL46	SLC25A1	SMARCA5
TPM3	MRPL49	SLC38A1	SMARCC2
TPM4	MRPS27	SPTLC1	SMC1A
TRAM1	MRPS31	SPTLC2	SMC2
TUBA1C	MTCH2	SQRDL	SMC3
UBA1	MTHFD1L	SRPR	SMCHD1
UGCGL1	MTX1	STARD3NL	STAG1
UTRN	MTX2	STOM	STK24
VARS	MYO6	STT3A	SUMF2
VASP	NCEH1	SYNGR2	SUMO2
VAT1	NDUFA10	TAP1	SUPT16H
VCL	NDUFA5	TAP2	TGFB1
VIL1	NDUFA9	TAX1BP3	TGFBI
VSNL1	NDUFB10	TCIRG1	TJP1
WASF2	NDUFS1	TGM6	TLN1
WDR1	NDUFS2	THBS1	TMED9
ZDHHC5	NDUFS3	THEM2	TMEM41A
	NDUFV1	TIMM44	TOMM40
	NIPSNAP1	TIMM50	TOP2A
	NOMO2	TMED1	TOR1A
	NSDHL	TMED5	TOR1AIP1
	NXF1	TMED7	TRIP12
	OAT	TMEM165	TRRAP
	OGDH	TMEM43	TUBG1
	OPA1	TNC	UACA
	OSBPL3	TOMM70A	UBTD1
	PC	TSPAN14	UHRF1
	PCYOX1	TUFM	UNC84B
	PDHA1	TXNDC3	UTRN
	PDPR		VIM
	PECR		WAPAL
	PEX13		WASL

HCV		SenV	
**Enter MAM**	**Leave MAM**	**Enter MAM**	**Leave MAM**
	PHB	VAMP3	WIZ
	PHB2	YARS2	WWC2
	PICALM		XRCC6
	PITRM1		ZMYM3
	PKP2		ZNF828
	PLXNB2		
	PMPCB		
	POFUT2		
	POLDIP2		
	POLRMT		
	PON2		
	PPA2		
	PRDX3		
	PROM1		
	PSAP		
	PSEN1		
	PSIP1		
	PTCD3		
	PTGES2		
	PTPRF		
	PTTG1IP		
	PVRL2		
	PYCR2		
	QSOX2		
	RAB1B		
	RAB6C		
	RDH13		
	RETSAT		
	RG9MTD1		
	RHOT2		
	RPN1		
	RPN2		
	SACM1L		
	SARS2		
	SCAMP2		
	SCAMP3		
	SDF4		
	SDHA		
	SEC11A		
	SEC62		
	SERINC1		

HCV		SenV	
Enter MAM	Leave MAM	Enter MAM	Leave MAM
	SFXN1		
	SHMT2		
	SLC1A5		
	SLC25A1		
	SLC25A11		
	SLC25A13		
	SLC25A24		
	SLC25A5		
	SLC35B2		
	SLC38A2		
	SLC4A11		
	SLC4A7		
	SMARCC1		
	SMARCC2		
	SMARCD2		
	SMPD4		
	SOD2		
	SPTLC1		
	SQRDL		
	ST6GAL1		
	STOML2		
	STX12		
	SURF4		
	TBL2		
	TBRG4		
	TFAM		
	TFRC		
	TJP1		
	TM9SF1		
	TM9SF4		
	TMED10		
	TMEM111		
	TMEM126A		
	TMEM176B		
	TMEM43		
	TOMM40		
	TOMM70A		
	TOP2A		
	TOP2B		

HCV		SenV	
**Enter MAM**	**Leave MAM**	**Enter MAM**	**Leave MAM**
	TRAP1		
	TSFM		
	TSPAN13		
	TST		
	TUFM		
	UQCRB		
	UQCRC1		
	UQCRC2		
	VDAC1		
	VDAC2		
	VDAC3		
	WFS1		
	XPNPEP3		
	YME1L1		
